# Method for Horizontal Calibration of Laser-Projection Transnasal Fiberoptic High-Speed Videoendoscopy

**DOI:** 10.3390/app11020822

**Published:** 2021-01-17

**Authors:** Hamzeh Ghasemzadeh, Dimitar D. Deliyski, Robert E. Hillman, Daryush D. Mehta

**Affiliations:** 1 “Department of Communicative Sciences and Disorders” and “Department of Computational Mathematics Science and Engineering”, Michigan State University, East Lansing, Michigan, USA; 2 “Department of Communicative Sciences and Disorders”, Michigan State University, East Lansing, Michigan, USA; 3 “MGH Institute of Health Professions”, “Center for Laryngeal Surgery and Voice Rehabilitation, Massachusetts General Hospital”, “Department of Surgery, Harvard Medical School”, and “Speech and Hearing Bioscience and Technology, Division of Medical Sciences”, Harvard Medical School, Boston, MA, USA; 4 “MGH Institute of Health Professions”, “Center for Laryngeal Surgery and Voice Rehabilitation, Massachusetts General Hospital”, “Department of Surgery, Harvard Medical School”, and “Speech and Hearing Bioscience and Technology, Division of Medical Sciences”, Harvard Medical School, Boston, MA, USA

**Keywords:** Horizontal calibrated measurements, Image distortion, Laser calibration, Flexible endoscopy, Laser projection endoscope, High-speed videoendoscopy, Instrumental voice assessment

## Abstract

**Objective:**

Calibrated horizontal measurements (*e*.*g*., mm) from endoscopic procedures could be utilized for advancement of evidence-based practice and personalized medicine. However, the size of an object in endoscopic images is not readily calibrated and depends on multiple factors, including the distance between the endoscope and the target surface. Additionally, acquired images may have significant non-linear distortion that would further complicate calibrated measurements. This study used a recently developed in-vivo laser-projection fiberoptic laryngoscope and proposes a method for calibrated spatial measurements.

**Method:**

A set of circular grids were recorded at multiple working distances. A statistical model was trained that would map from pixel length of the object, the working distance, and the spatial location of the target object into its mm length.

**Result:**

A detailed analysis of the performance of the proposed method is presented. The analyses have shown that the accuracy of the proposed method does not depend on the working distance and length of the target object. The estimated average magnitude of error was 0.27 mm, which is three times lower than the existing alternative.

**Conclusion:**

The presented method can achieve sub-millimeter accuracy in horizontal measurement.

**Significance:**

Evidence-based practice and personalized medicine could significantly benefit from the proposed method. Implications of the findings for other endoscopic procedures are also discussed.

## 1. Introduction

The ability to perform measurements is an important cornerstone and prerequisite of any quantitative research. Measurements allow us to quantify inputs and outputs of a system, and then to express their relationships using concise mathematical expressions and models. Those models would then enable us to understand how a target system works and to predict its output for changes in the system parameters. Conversely, models could be utilized to determine the proper parameters of a system for achieving a certain output. Putting these in the context of voice science research, variations in the parameters of the phonatory system could be attributed to individual differences. Thus, accurate models would enable us to account for individual differences during the diagnosis and to make reliable predictions about the likely outcome of different treatment options. Analysis of the vocal fold vibration using high-speed videoendoscopy (HSV) could be an ideal candidate for constructing computational models [1–3]. Such patient-specific models could advance personalized medicine in the fields of laryngology and speech-language pathology. Additionally, previous works have suggested vibratory characteristics and kinematic measures from laryngeal videos can be utilized for direct evaluation of treatment outcome [4,5]. Reduction in post-treatment lesion size could be another objective measure for direct evaluation of a treatment outcome. Realization of such direct evaluation criteria could provide the required scientific evidence behind the efficacy of different interventions, and answer a recent call for evidence-based practice in the clinical assessment of voice [6,7]. Performing spatially calibrated measurements could provide significant benefit for these applications. Accurate mapping of laryngeal diseases [8], studying the developmental aspects of vocal folds [9], the sex differences in the vocal fold morphology [10,11], and the effects of singing on the morphology of the vocal folds [12,13], are just few other examples among many possible applications that would significantly benefit from calibrated spatial measurements.

Unfortunately, conventional images cannot be used for calibrated spatial measurements. Specifically, multiple factors may act as confounding variables for the length of an object in its image. To make a distinction between the actual length of an object and its length on the image, they will respectively be called mm length and pixel length for the rest of this paper. Everybody has experienced the inverse relationship between the pixel length of an object and its distance from the camera. Furthermore, the imaging system may exhibit specific non-linear distortions such that the pixel length of an object would depend on its spatial location [14]. Due to such factors, we cannot readily measure the mm length of an object from its pixel length. However, the existence of some *auxiliary* information on the images could alleviate this. The *auxiliary* information could come from some properly designed fiducial markers that are projected to the field of view (FOV). Laser source emits spatially coherent light and therefore can be used for creating fiducial patterns with specific topological properties. The created pattern could then be delivered by clipping the laser projection component to an endoscope [9,15–19], or by employing a surgical endoscope [20]. It is noteworthy that optical coherence tomography is another imaging modality that could provide calibrated measurement capabilities [21–23].

Two main approaches of parallel laser markers and multiple laser points have been used for creating the laser-fiducial markers in the field of voice [24]. The projection of the parallel laser markers is the simplest approach. Two-point laser projection [15,16,25], two-line laser projection [26], and multiple line laser projection [9] are some examples of this category. The multiple-laser-points projection is more sophisticated and involves the projection of many laser points on the FOV [8,20,27,28]. Each method has its own merits. The parallel laser projection category benefits from the simplicity of its optical design and subsequent measurement methodology. Detection of the laser markers on the image is the only required step for measurement in those systems. After that, the distance between the laser markers may be used similarly to a scale on a map and calibrated horizontal measurements may be achieved using a simple caliper. The main assumption of this method is that all pixels in the image have the same mm sizes which could be violated if different objects of the image have different distances from the camera, or if different locations of the image have different pixel size representations [14]. Violation of this assumption will lead to measurement errors. Conversely, multiple-laser projection systems benefit from the presence of laser points in all parts of the image. Not only this information helps with vertical measurements [24] and 3D reconstruction of the envelope of the FOV [19], but it also means that with a high probability some laser points would be near to, or on the target surface. Therefore, the above-discussed problems would be resolved. However, systems from this category require more sophisticated optical hardware and processing software design. Horizontal measurements from these systems depend on a calibration step, where the confounding factors of pixel-to-mm conversion scale are determined and accounted for.

The main purpose of this paper is to present the method for horizontal calibration and subsequent horizontal measurements using a recently-developed multiple-laser point transnasal fiberoptic endoscope [20]. The rest of this paper is organized as follows. The Materials and Methods section presents details of the algorithm and the recordings used for development and evaluation of the proposed method. The Experiments and Results section consists of four experiments that evaluate the performance of the algorithm in different conditions and scenarios, followed by relevant discussions of the results. The implications of the results are put into research and clinical contexts in the Discussion section.

## 2. Materials and Methods

**Figure 1.**
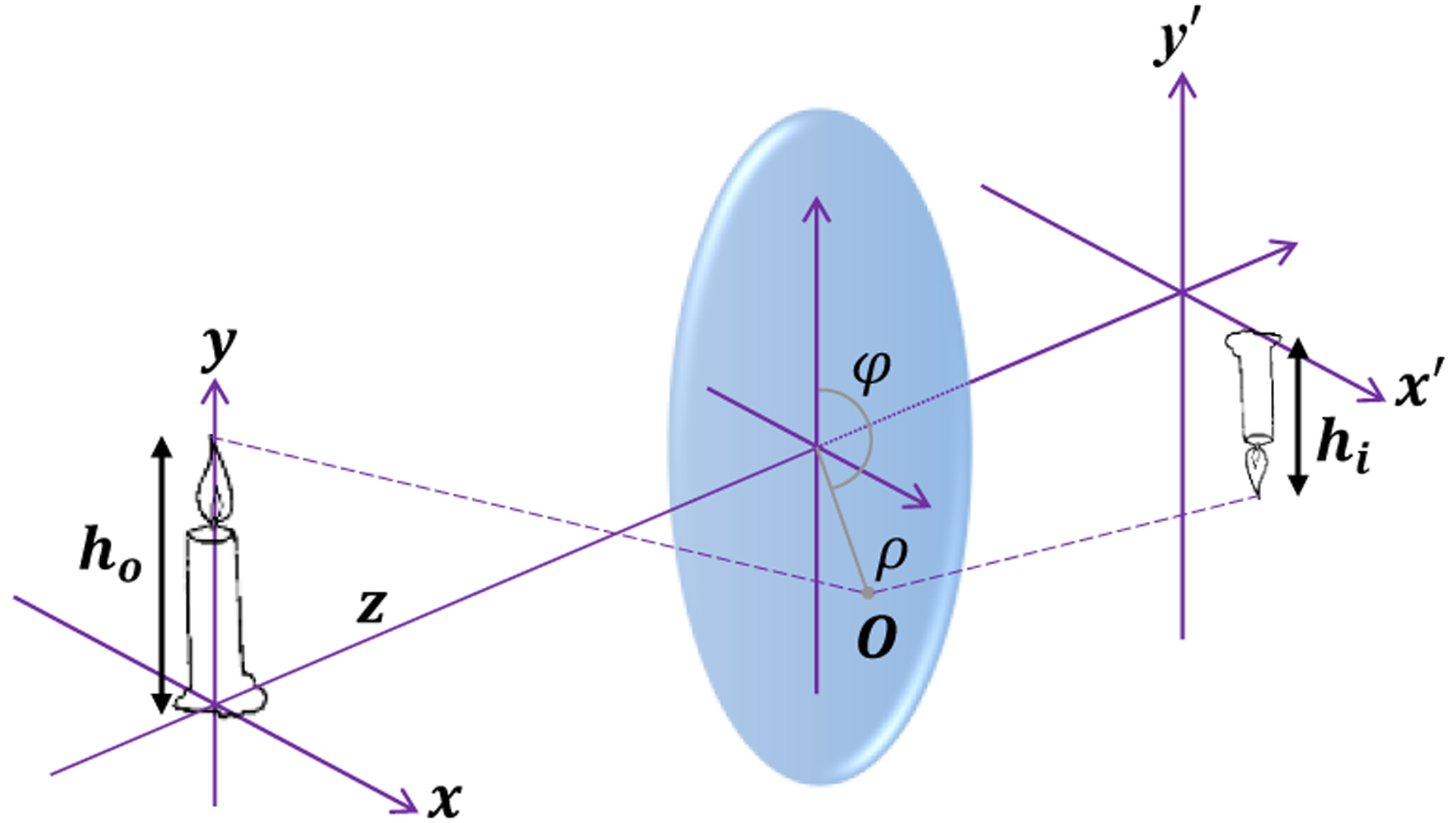
Relationship between the length of an object (*h*_*o*_) and its image (*h*_*i*_) in an axially symmetrical optical

Assuming a perpendicular imaging angle, the mm length of an object can be measured accurately. Accurate estimation of the mm length of an object from the pixel length of the object’s image is the primary goal of calibrated horizontal measurement. Assuming an optical system that is symmetrical around its optical axis, the relationship between an object and its image can be determined. Let *h*_*o*_ denote the mm length of an object and *h*_*i*_ denote the pixel length of the object’s image. Also, let O be the intersection point between a ray of light from the object and the lens of the camera (Figure 1) expressed in the polar coordinates (*ρ*, *φ*). We would have [29],

(1) $$h_{i}=A_{1}\rho \text{cos}\varphi +A_{2}h_{0}+B_{1}\rho^{3}\text{cos}\varphi +B_{2}\rho^{2}h_{0}(2+\text{cos}2\varphi )+\left(3B_{3}+B_{4}\right)\rho h_{o}^{2}\text{cos}\varphi +B_{5}h_{o^{+}}^{3}+\text{HOT}$$

where *A*_*j*_ and *B*_*j*_ are constants, and HOT represents the higher-order terms. Readers interested in more detailed analysis of image formation may refer to [29] and its references.

Equation 1 shows a non-linear and complex relationship between pixel and mm lengths. Using the thin-lens assumption and small-angle approximation [29] Equation 1 can be approximated with a much simpler model known as the Gaussian optics [29]. In this model, the ratio of pixel length to mm length is a constant number, which is called the magnification factor of the system (*m*). Equation 2 shows this:

(2) $$m=\frac{h_{i}}{h_{o}}$$

In the Gaussian optics, magnification factor and the working distance have an inverse relationship [29], and therefore the working distance would be a confounding factor for calibrated horizontal measurements.

Flexible fiberoptic endoscopes employ wide-angle lenses to maximize their FOV sizes. However, wide-angle lenses violate the small-angle approximation of the Gaussian optics. This leads to a more complex relationship between pixel and mm lengths. Specifically, this deviation introduces significant non-linear distortion into recorded images. Recently, Ghasemzadeh et al. [14] studied the distortion of a flexible laryngoscope and showed that when the imaging axis is perpendicular to the target surface, the distortion is symmetrical around the optical axis, and points with similar distances from the FOV center experience similar distortions. Considering this symmetry, Equation 1 may govern the image formation in flexible endoscopy. Additionally, that study showed the pixel length of an object significantly depends on its spatial location within the FOV [14]. Therefore, the spatial location of the target object is another confounding factor for horizontal measurements. Circular grids can exploit this symmetry efficiently; thus, the proposed method uses circular grids to account for the effect of working distance and the spatial location of the target object.

**Figure 2.**
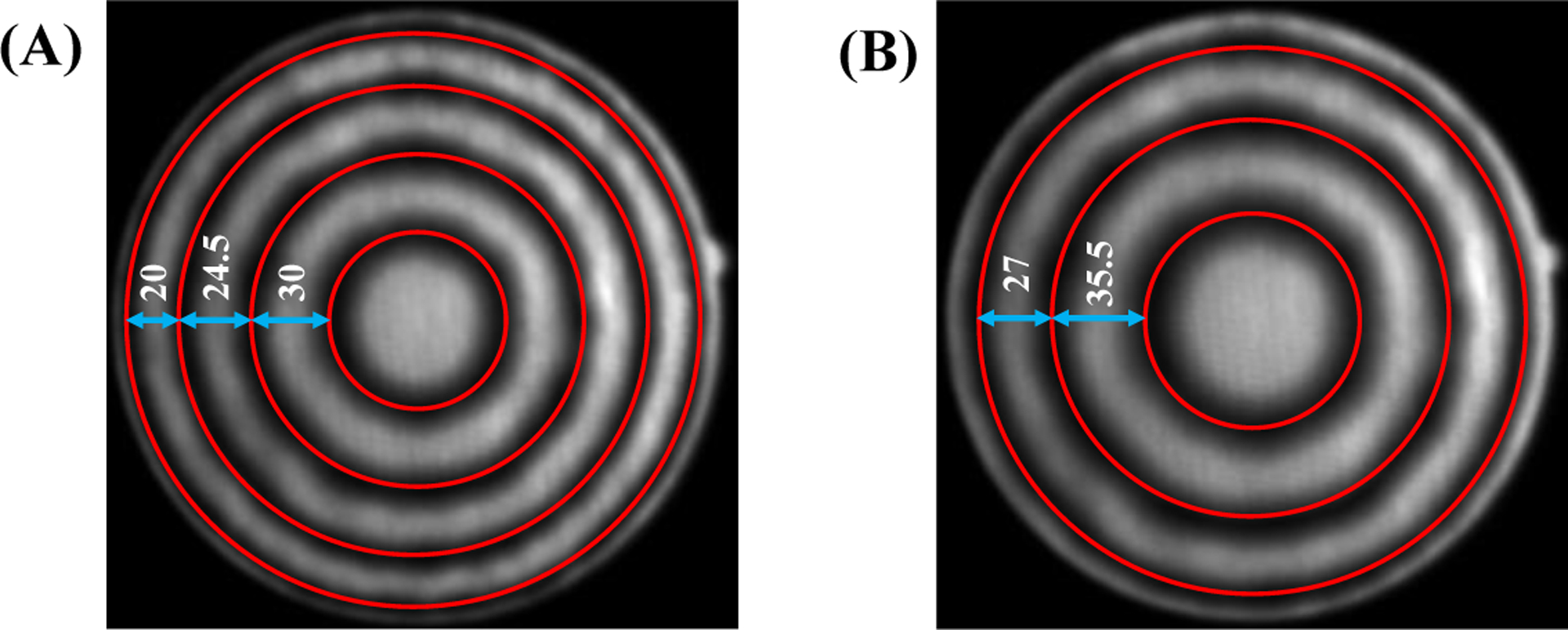
Effects of working distance and spatial location on horizontal measurements: **(A)** working distance of 2.87 mm, **(B)** working distance of 2.24 mm.

To demonstrate this, a circular grid with the spacing of 0.5 mm was recorded at working distances of 2.87 mm and 2.24 mm. Figure 2 shows the recorded images. The circles had a constant distance of 0.5 mm from each other. However, in fig 2–A we see as we go from the center toward the periphery the distance between consecutive circles decreases from 30 pixels to 20 pixels. This clearly demonstrates the dependence of horizontal measurements on the spatial location. Comparing fig 2–A and 2–B we see the effect of working distance, where the distance between the two smallest circles increases from 30 pixels to 35.5 pixels when the working distance decreases from 2.87 mm (fig 2–A) to 2.24 mm (fig 2–B).

### 2.1. Recording instrumentation and setup

The data acquisition system consisted of a custom-built laser-projection flexible endoscopy system [20] attached to a high-speed monochrome Phantom v7.1 camera (Vision Research Inc., Wayne, NJ). The laser-projection endoscope was based on the surgical fiberoptic endoscope, Fiber Naso Pharyngo Laryngoscope Model FNL-15RP3 (PENTAX Medical, Montvale, NJ). The surgical channel of the endoscope was used for housing of the optical components of the laser projection system and delivering the laser pattern on the FOV. A diffraction-based design was used to create a 7×7 grid pattern from a 520 *nm* green-laser beam [20].

The proposed calibration and subsequent horizontal measurement methods were developed and then evaluated based on different sets of benchtop recordings. The employed setup consisted of an adjustable arm for precise tuning of the distance between the distal tip of the endoscope and the target surface (*i*.*e*., working distance) [14, 24]. A digital height gauge with an accuracy of 0.001″ (0.025 mm) was used for measurement of the working distance. All recordings were carried out at a spatial resolution of 288×280 pixels and speed of 100 frames per second. Considering that images were taken from static surfaces, this frame rate was irrelevant, where 100 fps was an adequate rate for our purpose [14].

### 2.2. Datasets

This study used four different sets of recordings. Set 1 contained 65 recordings from circular grids (Figure 2) at different working distances. This set was used for training and testing of the model converting a pixel length to its mm length. The working distance was gradually increased from 2 mm to 32 mm. This range covers the working distances applicable to laryngeal flexible endoscopy. At each working distance, a recording was done. This process was repeated three times to reduce measurement error. For each recording, the grid was adjusted subjectively inside the FOV such that the largest visible circle had a uniform distance from the border of the FOV. Considering the limited spatial resolution, grids became significantly blurry after a certain working distance. Hence, three different circular grids with the spacing of 0.5 mm, 1 mm, and 2 mm were used for working distances in the range of [2, 10], [10, 20], [20, 32] mm. The presence of the laser points was saturating some of the black pixels belonging to the grids. This could affect accurate detection of the grid. Also, the laser points were not necessary for the purpose of this set; therefore, the laser source was turned off during these recordings.

The proposed method requires an accurate estimation of the distance between the tip of the endoscope and the target surface (*i*.*e*., the working distance). Previously, it was shown that a statistical model can be trained to decode the working distance from locations of the laser points [24]. Set 2 had 72 recordings and was used for the training of the model that estimates the working distance. For this set, the laser source was turned on, and the light source was turned off and recordings were done from a white paper. The working distance was gradually increased from 2 mm to 35 mm and at each working distance, a recording was done. The recording process was repeated four times to reduce measurement error.

**Figure 3.**
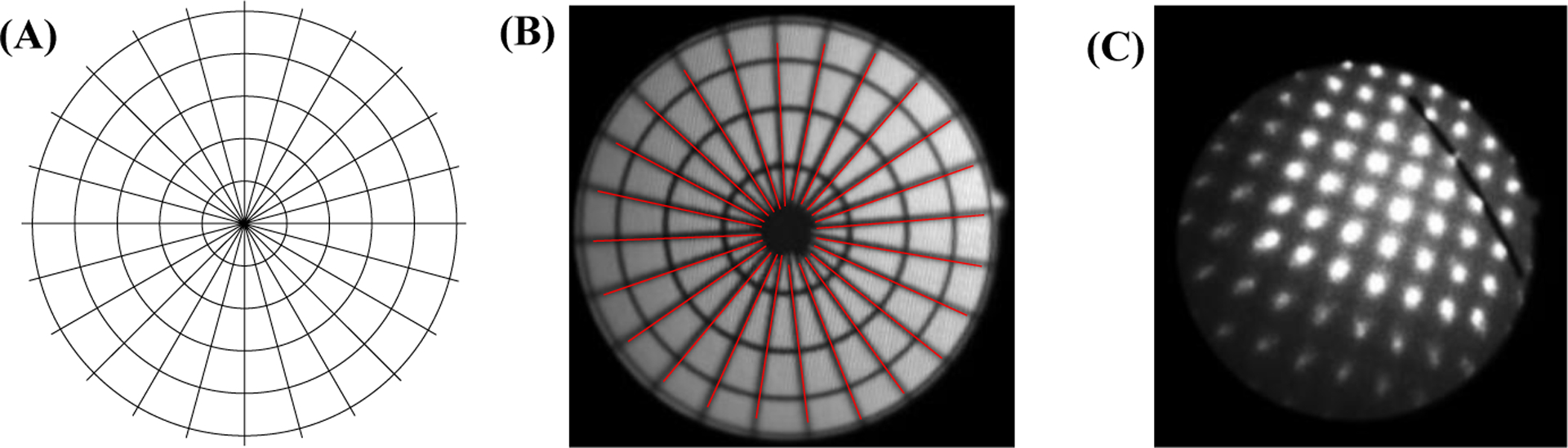
The data for evaluation of central angle measurement: **(A)** the custom-designed grid, **(B)** segmented radial lines, (C) an example of a line segment with the projected laser points.

The proposed method relies on an accurate estimation of a central angle (*i*.*e*., an angle that has its apex on the center of a circle). However, flexible endoscopy images exhibit significant nonlinear distortions [14]. Set 3 was recorded to investigate possible effects of the introduced nonlinear distortion on central angle measurements. This set was based on a custom-designed grid. A circular grid was divided into 24 equal sectors, which created 24 central angles in 15° increments (Figure 3–A). The grid was recorded at four working distances of 6.16 mm, 13.20 mm, 19.54 mm, and 26.44 mm. At each recording distance, the grid was adjusted subjectively inside the FOV such that the largest visible circle had a uniform distance from the border of the FOV. This process insured that the center of the grid was at the center of the FOV. This characteristic governs that estimated angles from the image are central angles. The laser source was turned off during these recordings.

Set 4 was recorded for evaluating the accuracy of the proposed method. Line segments with known mm lengths were recorded at fifteen arbitrary locations in the FOV with arbitrary rotations. To provide a comprehensive evaluation, a wide range of lengths and working distances were used. Specifically, 5 mm, 10 mm, 15 mm, and 20 mm line segments were recorded at a working distance of 20.18 mm. These recordings were used to investigate the possible effect of object length on the accuracy of the method. Additionally, a 5 mm line segment was recorded at working distances of 5.12 mm, 9.98 mm, 14.98 mm, and 20.18 mm, which covers the common range of administration of fiberoptic laryngeal endoscopy. These recordings were used to investigate the possible effect of working distance on the accuracy of the method. The laser source was turned on during these recordings. Figure 3(C) presents an example from Set 4.

Table 1 presents a summary of each data set.

### 2.3. Segmentation and preprocessing

**Figure 4.**
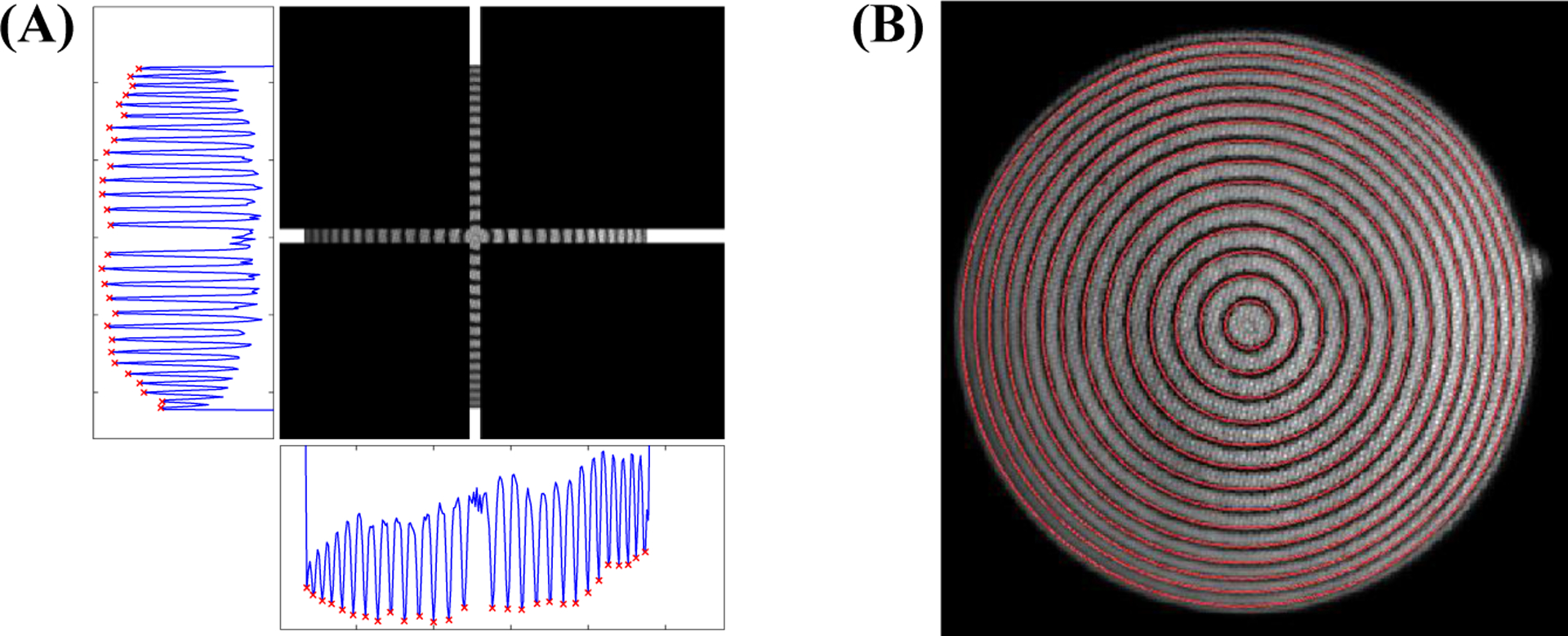
Segmentation of a circular grid: **(A)** strips parallel to axes with their respective summations (the red × marks denote the detected local minima), **(B)** final segmented circles after the fine-tuning stage.

Accurate detection of circular grids is a prerequisite of the proposed calibration method. An automatic two-stage method was developed for the segmentation of the circles from Set 1. To take advantage of the full 72-dB dynamic range of the camera, recordings were imported into MATLAB (MathWorks, Natick, MA) directly in the native 12-bit format from the proprietary Vision-Research .cine (Vision Research Inc., Wayne, NJ, USA) files without any conversion or compression. To reduce the noise of images, frames of the recordings were averaged over time and then a Gaussian filter with a size of 2 pixels was applied. The Center and radius of the FOV were estimated using the method described in [24]. A strip parallel to the *x*-axis centered at the center of the FOV with a width of 9 pixels was selected. The strip was averaged over the rows, and then locations of its local minima were detected. Detected locations were paired based on their distances from the center of the FOV. The average of each pair was used as the coarse estimation of the *x*-coordinate of centers of circles. Half of the difference between each pair was used as the row-wise estimation of radii of circles. This process was repeated for a strip parallel to the *y*-axis averaged over the columns. The average of each pair was used as the coarse estimation of the *y*-coordinate of centers of circles. Half of the difference between each pair was used as the column-wise estimation of radii of circles. The final coarse estimation of the radius of each circle was computed as the average of its row- and column-wise radii. A grid search over all combinations of the three estimated parameters ±1 pixel with the resolution of 0.25 pixels was used for fine-tuning of the estimated parameters. Specifically, for each case, the target parameters were used to create a ring mask with a width of 1 pixel. The mask was then applied to the gradient of the image, and the summation of the results was used as the cost function. The set of parameters that minimized the cost function was selected as the final estimation of the center and radius of each circle. Figure 4 shows the process, with the results on an example image.

The segmentation of the laser points for Set 2 was based on the method described in [24]. Target objects in Set 3 were 24 radial lines, which were detected using the Hough transform [30]. Figure 3–B shows the grid after segmentation. The actual horizontal measurements on laryngeal images will rely on the manual segmentation of the target object. To better reflect this, a graphical user interface was developed for manual segmentation of line segments from Set 4.

### 2.4. Horizontal calibration method

Working distance and spatial location of the target object are the main confounding factors of horizontal measurements. Circular grids provide an effective way for the spatial sampling of the location inside the FOV. This information can be utilized for determining the dependence of horizontal measurements on the spatial location. Additionally, the grids can be recorded at multiple working distances. This information may be utilized for determining the dependence of horizontal measurements on the working distance. To that end, all circles from Set 1 were segmented. Obviously, depending on the working distance different numbers of circles will fall inside the FOV, and hence will be recorded. The segmentation process of all 65 recordings resulted in 612 different data points. Let *w*, *r*_*p*_(*w*), and *r*_*mm*_(*w*) denote the working distance, pixel radius, and mm radius of a circle, respectively, recorded at *w* mm. Then, a statistical model can be trained using *w* and *r*_*p*_ as the predictor variables and *r*_*mm*_ as the outcome variable. Let $F_{M,N}$, denote a polynomial model in two variables, *w* and *r*_*p*_, with maximum degrees of *M* and *N*, respectively. Equations 3,4 show the model, where *a*_*l*,*k*_ are some constants determined during the training process:

(3) $$r_{mm}=F_{M,N}\left(w,r_{p}\right)$$

(4) $$F_{M,N}\left(w,r_{p}\right)=\sum_{k=1}^{M}\sum_{l=1}^{N}a_{l,k}\cdot w^{k}\cdot r_{p}^{l}$$

**Figure 5.**
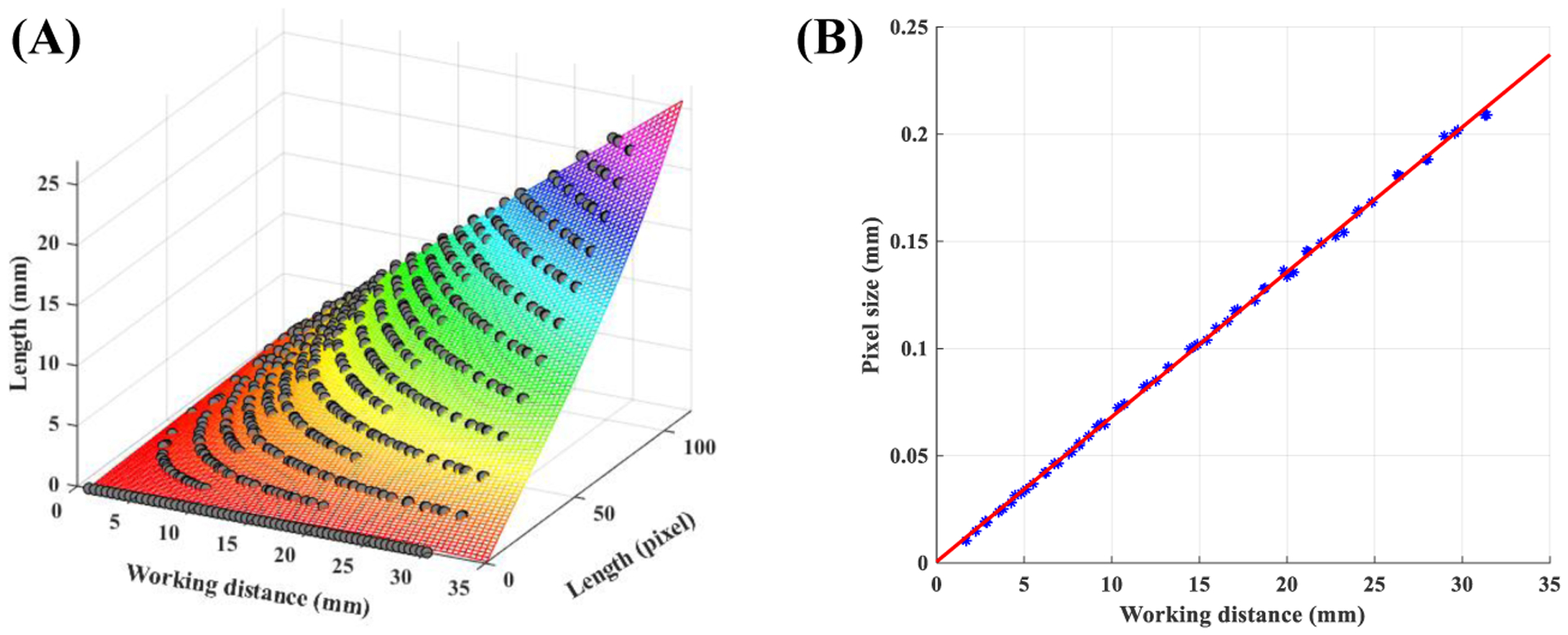
Models for horizontal measurements: **(A)** non-uniform model and **(B)** uniform model.

To select the best model, polynomial models with different degrees were evaluated using 10-fold cross-validation. The cost function was defined as the mean absolute error (MAE) over all testing samples from all 10 folds. The $F_{5,5}$ resulted in the MAE of 0.025 mm, which was the lowest value. This model will be referred to as the non-uniform model in the rest of this paper. Figure 5–A presents the trained non-uniform model.

To highlight the effect of spatial location on horizontal measurements, a second model was trained where all pixels in the FOV had similar pixel sizes. This scenario mimics horizontal measurement from a parallel-laser projection system. Let, *R*_*p*_(*w*) and *R*_*mm*_(*w*) denote the pixel radius and mm radius of the largest circle visible in the FOV recorded at the working distance of *w* mm. The uniform pixel size *γ*(*w*) is defined as

(5) $$\gamma (w)=\frac{R_{mm}(w)}{R_{p}(w)}.$$

The uniform pixel size was computed for all recordings in Set 1. Then, a statistical model was trained using the working distance as the predictor variable and *γ* as the outcome variable. Investigating the relationship between working distance and *γ* revealed a linear model. This model is shown in Figure 5–B and it will be referred to as the uniform model in the rest of this paper.

### 2.5. Horizontal measurement method

The application of the uniform model is simple and quite similar to the estimation of a distance on a printed map. The pixel size (*γ*) allows the conversion from pixel length into mm length. Considering the dependence of *γ* on the working distance, the following steps were followed for horizontal measurements using the uniform model. The working distance was estimated from the positions of the laser points [24]; then the appropriate value of the pixel size was computed from the uniform model. The pixel length of the target object was measured on the image; then the pixel length was multiplied with the multiplicative factor of pixel size to estimate its mm length.

The application of the non-uniform model is more involved, and it is described under two categories of radial and general measurements. A radial measurement is defined as the length of an object that has one of its ends on the FOV center. The non-uniform model was trained using circles centered at the FOV center. Therefore, the model can estimate the mm radius of a circle centered at the FOV center, which would be equivalent to a radial measurement. Thus, the following steps were followed for horizontal radial measurements using the non-uniform model. The working distance was estimated from the positions of the laser points [24]. Then, the pixel length of the target radial object was measured on the image. The values of working distance and pixel length were fed into the trained non-uniform model (eq. 3), and the mm length of the object was estimated.

**Figure 6.**
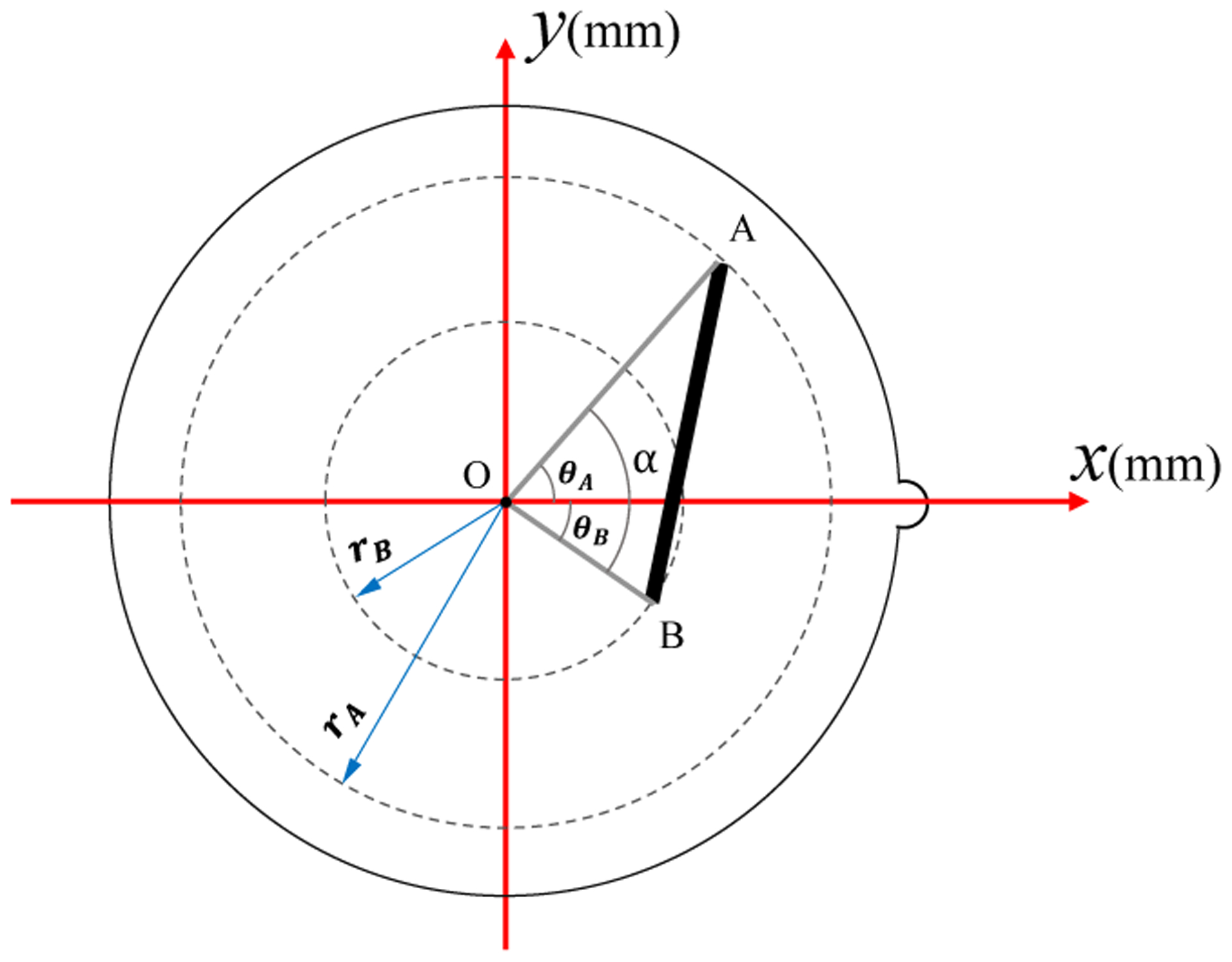
Expressing a general measurement in terms of radial measurements.

A general measurement needs to be expressed in terms of radial measurements before the application of the non-uniform model. Figure 6 shows this process. The main goal is to determine the length of the line segment AB in mm. We can construct the triangle AOB on the image, where O is the FOV center. Referring to Figure 6, OA and OB each has one of their ends at the FOV center, and hence they constitute radial measurements, and their mm lengths can be computed using the non-uniform model. At the same time, we can measure the angle α from the image. Let $A|{}_{y_{A}}^{x_{A}}$ and $B|{}_{y_{B}}^{x_{B}}$ be on the image then, we can determine the angle between OA and the positive *x*-axis (*θ*_*A*_) as follows,

(6) $$\theta_{A}={\text{tan}}^{-1}\left(\frac{y_{A}}{x_{A}}\right)$$

where *tan*^−1^ denotes the four-quadrant inverse tangent function. The angle between OB and the positive *x*-axis (*θ*_*B*_) can also be measured, similarly. Finally, the angle *α* can be computed as,

(7) $$\alpha =\left|\theta_{A}-\theta_{B}\right|$$

Now, we can apply the law of cosines for determining the mm length of the line segment AB:

(8) $${\text{AB}}^{2}={\text{OB}}^{2}+{\text{OA}}^{2}-2\cdot \text{OA}\cdot \text{OB}\cdot \text{cos}(\alpha )$$

### 2.6. Estimation of the working distance

Referring to Equations 3 and 5, we see that accurate estimation of the working distance is a prerequisite of both uniform and non-uniform methods. The method for estimating the working distance has been presented in [24]. The method is described very shortly here, followed by an improvement over the previous approach. The position of a laser point is a function of the working distance, once the effect of rotation and displacement of the FOV are compensated for [24]. Therefore, we may train a statistical model that could decode the working distance from the position of a laser point. In [24], this was achieved by converting the position of the laser point from the Cartesian coordinate system into the polar coordinate system. Then, the radius component (*r*) of the position of the laser point was used for the training of the model. Equations 9, 10 show the model (G), where *r*_*ij*_ is the radius computed from the laser point *i* at the working distance *w*_*j*_, and *β*_*i*1_, *β*_*i*2_, *β*_*i*3_, *β*_*i*4_ are some constants determined during the training process:

(9) $$w_{j}=G\left(r_{ij}\right)$$

(10) $$G\left(r_{ij}\right)=\beta_{i1}e^{\beta_{i2}r_{ij}}+\beta_{i3}e^{\beta_{i4}r_{ij}}$$

The original model also assumed that the data were mapped into a standard template by applying a chain of rotation, translation, and scaling operations on the recorded images. Flexible endoscopes have a fiducial marker (see, *e*.*g*., protrusion on right side of circle in Figures 4 and 6) that helps with the orientation of the recorded images. The rotation operation was parametrized in terms of the angle between the positive *x*-axis and the line connecting the fiducial marker to the FOV center. The rotation operation brings this angle to a fixed and standard value across all recordings [24]. This value will be further termed standard angle. In essence, the standard angle it is the angle between the fiducial marker and the *x*-axis of the image after undergoing the rotation operation. For example, the standard angle of Figure 6 is zero degrees. First, we show that the performance of the original method depends on the value of this angle; then we propose an improved version to alleviate this problem.

**Figure 7.**
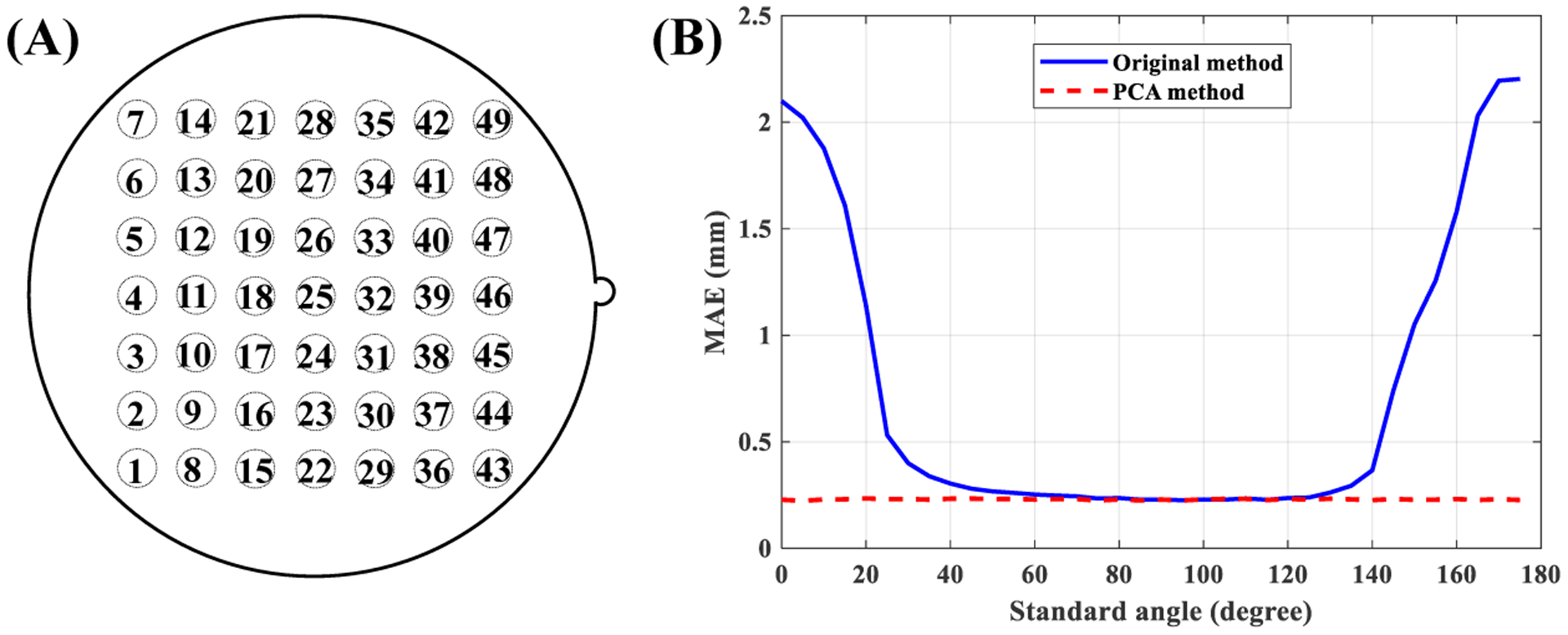
**(A)** indexing of the laser points, **(B)** mean absolute error (MAE) of original and the proposed PCA method for different values of standard angle.

Ten-fold cross-validation over Set 2 was used to evaluate the effect of different standard angles on the accuracy of estimation of the working distance. To that end, the standard angle of the method proposed in [24] was varied between 0° and 180° in 5° increments, and then the segmented laser points from the training set were used to create the model. It has been shown that laser points from the top row (Figure 7–A) degrade the accuracy of measurements [24]; therefore laser points from the top row were discarded for this analysis. The trained model was then applied to the testing set, and measurement errors from the remaining 42 laser points were computed. MAE over all 10 folds is shown in Figure 7–B.

Investigation of Figure 7–B shows that the accuracy of the original method highly depends on the choice of the standard angle. Principal component analysis (PCA) is a mapping that is robust to linear transformations of the data points, including their rotation. Consequently, we propose a slight improvement over the original method. Let $p_{ij}|{}_{y_{ij}}^{x_{ij}}$ be the cartesian coordinates of the laser point *i* (1≤*i*≤49) at the working distance *w*_*j*_. We can store *p*_*ij*_ for a specific value of *i* and all values of *j* (1≤*j*≤*n*) into a 2×*n* data matrix P_i_, where *n* is the number of working distances in the dataset. Let *μ*_*ix*_ and *μ*_*iy*_ denote the average values of *P*_*i*_ over the first and the second row, respectively. Now, we can center the data and construct the matrix *Q*_*i*_. Equations 11–13 show these definitions. **1**_*n*_ is a column vector containing 1 in all of its *n* rows.

(11) $$\mu_{ix}=\frac{\sum_{j=1}^{n}x_{ij}}{n}$$

(12) $$\mu_{iy}=\frac{\sum_{j=1}^{n}y_{ij}}{n}$$

(13) $$Q_{i}=P_{i}-\left[\begin{array}{l} \mu_{ix}\\ \mu_{iy} \end{array}\right]\cdot 1_{n}^{T}$$

Now the direction capturing most of the variance of the data (***u***_*i*_) can be computed as,

(14) $$u_{i}=\underset{\|u\|=1}{\text{arg max}}\left(u^{T}\cdot Q_{i}Q_{i}^{T}\cdot u\right)$$

The first principal component (***v***_*i*_) would be the projection of the data points on the direction ***u***_*i*_ and is computed as,

(15) $$v_{i}=u_{i}^{T}\cdot Q_{i}$$

Now, the first principal component may be used to train the vertical calibration model. Let *v*_*ij*_ denotes the *j* component of the vector ***v***_*i*_ (*i*.*e*. projection of the point *p*_*ij*_ in direction ***u***_*i*_). Equations 16–17 are repeated for each laser point *i*.

(16) $$w_{j}=G_{i}\left(v_{ij}\right)$$

(17) $$G_{i}\left(v_{ij}\right)=\beta_{i1}e^{\beta_{i2}v_{ij}}+\beta_{i3}e^{\beta_{i4}v_{ij}}$$

Ten-fold cross-validation over Set 2 was used to evaluate the effect of different standard angles on the accuracy of the improved model. The standard angle was varied between 0° and 180° in 5° increments and for each value. The training set was used to estimate *μ*_*ix*_, *μ*_*iy*_, ***u***_*i*_, and parameters of the model (*β*_*i*1_, *β*_*i*2_, *β*_*i*3_, *β*_*i*4_). The trained model was then applied to the testing set and measurement errors were computed. Figure 7–B shows the computed MAE of the proposed method over all 10 folds. This figure shows the robustness of the improved method to variations in standard angle. Experiment 1 in the next section presents the performance of the proposed improved method in more detail.

## 3. Experiments and results

### 3.1. Experiment 1

**Figure 8.**
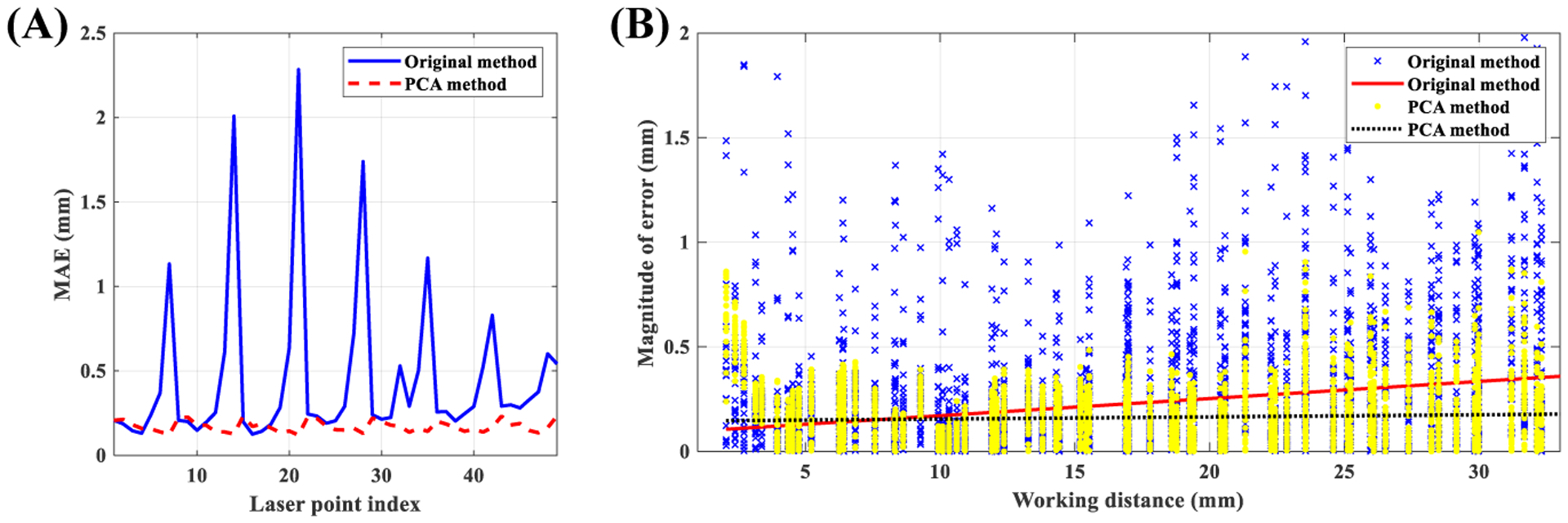
Performance of estimating the working distance: **(A)** measurement accuracy of different laser points, **(B)** effect of working distance.

This experiment was based on Set 2. The accuracy of the improved vertical measurement model (eq. 16–17) was compared with the original method [24] using 10-fold cross-validation. The original method used the value of 30° for the standard angle. At this angle, the grid becomes a square that is parallel to the *x*-*y* axis, which facilitates the labeling of the laser points. The same standard angle was also used for the proposed improved version. Recordings from Set 2 were split into training and testing sets. Both models were trained using the training set. The trained models were then applied to the testing set and measurement errors were computed. First, the effect of different laser points on the error was investigated. MAE was computed for each laser point averaged over all working distances. Figure 8–A shows the result. Based on this figure we see different laser points exhibit different performances in the original method, where the top-row laser points (refer to Figure 7–A) produce inferior results. Conversely, all laser points exhibit comparable performances in the improved PCA method. A second analysis was conducted to test the effect of working distance on the accuracy of both methods. For this analysis, the magnitude (i.e., absolute value) of error from laser points at different working distances were computed. Additionally, laser points from the top row were discarded from the original method and only the remaining 42 laser points were used. However, all 49 laser points were used for the analysis of the improved PCA method. Figure 8–B shows the results. The lines represent the linear model fitted on the individual data points. We can use the slope of regression lines to compare the dependence of the magnitude of error of different methods on the working distance. Slopes of original and PCA methods were 0.008 mm/mm and 0.001 mm/mm, respectively. Therefore, we may conclude that the performance of the improved PCA method is less dependent on the working distance.

### 3.2. Experiment 2

**Figure 9.**
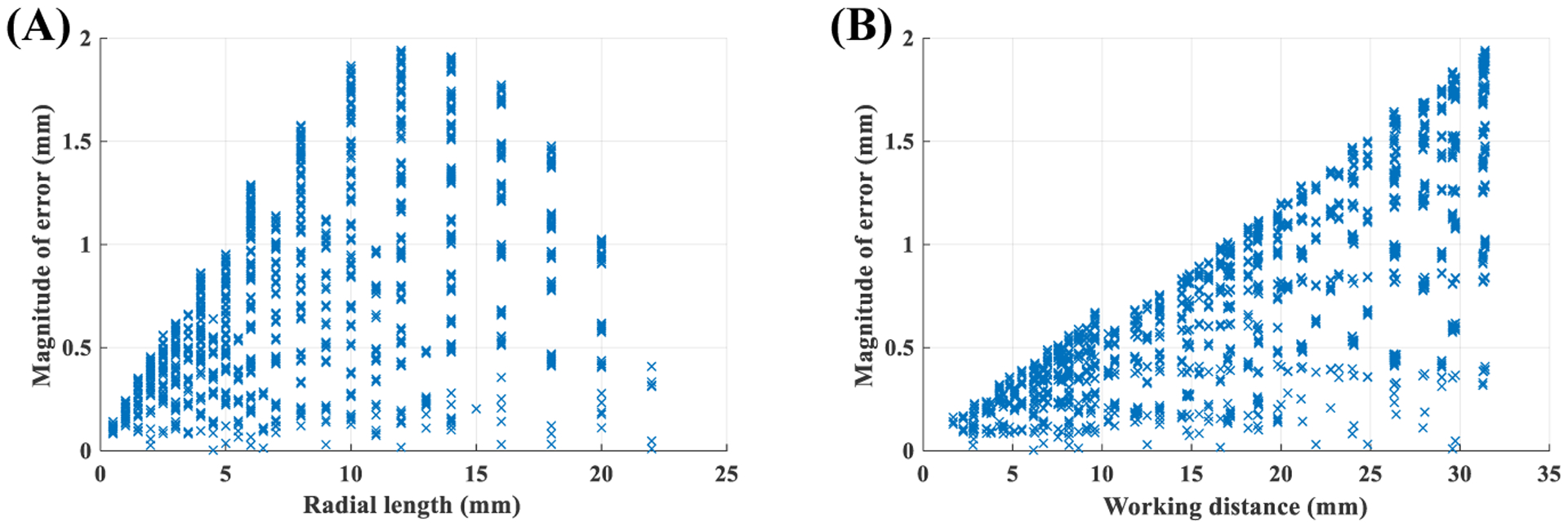
Performance of uniform model for radial measurements: **(A)** effect of object length, **(B)** effect of working distance.

This experiment was based on Set 1. The accuracy of the uniform model for radial measurement was evaluated using 10-fold cross-validation. To that end, Set 1 recordings were split into training and testing sets. The uniform model was trained using the largest enclosed circles of the training set. The trained uniform model was then evaluated for estimating mm radii of all circles from the testing set, in addition to smaller circles (those that were not used during the training process) of the training set. Figure 9 presents scatter plots of the magnitude of errors over all 10 folds relative to the radial length of the target circle and the working distance.

Investigating scatter plots of Figure 9 reveals that the measurement error of the uniform model depends on the working distance and the length of the target object. However, the relationship seems to be non-linear. Additionally, our analysis showed that neither of the variables had a normal distribution. Therefore, both parametric and non-parametric tests were used to quantify the effect of working distance and length of the object on the magnitude of the error. Table 2 reports values of Pearson’s *r*, Kendall’s *τ*, and Spearman’s *ρ*. Based on Table 2, we see a moderate positive correlation between the magnitude of error and length of the target object and a strong positive correlation between the magnitude of the error and the working distance.

**Figure 10.**
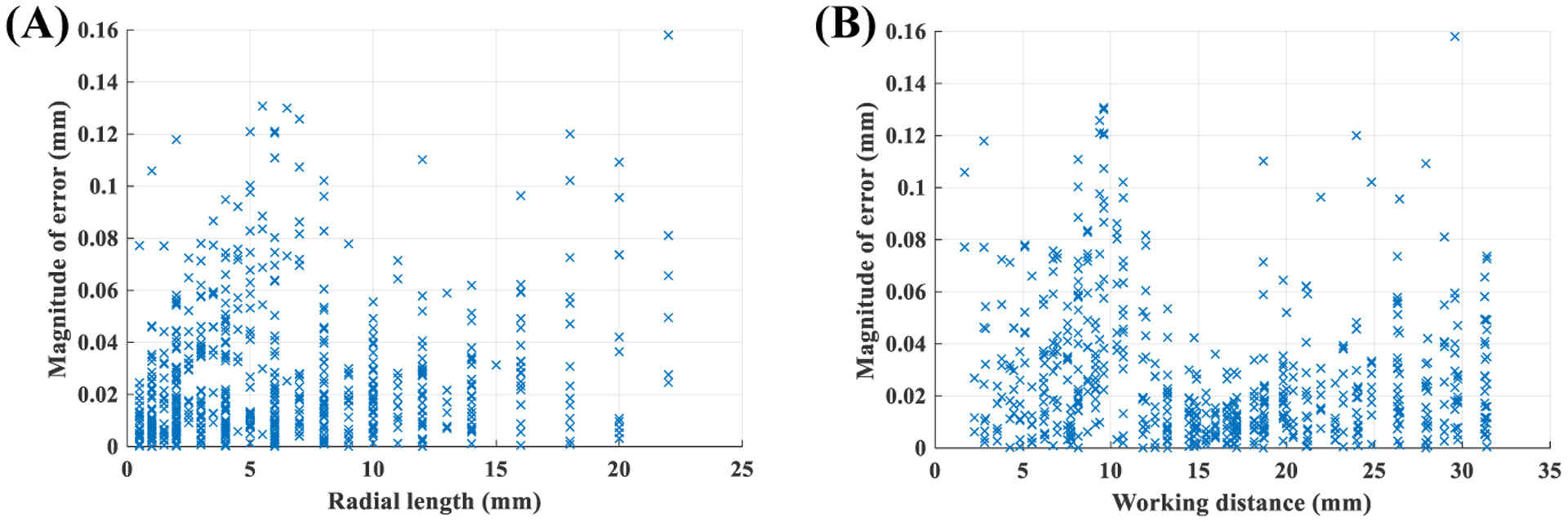
Performance of non-uniform model for radial measurements: **(A)** effect of object length, **(B)** effect of working distance.

The non-uniform model was trained using the training set, and then its performance for estimating the mm radii of circles was evaluated using the testing set. Figure 10 presents scatter plots of the magnitude of errors over all 10 folds relative to the radial length of the target circle and the working distance.

Table 3 quantifies the effect of radial length of the object and working distance on the magnitude of error from the non-uniform model. Based on Table 3, we see the magnitude of error has very week associations with the working distance and length of the target object.

Comparing results of tables 2 and 3 highlights a primary advantage of the non-uniform method over its uniform counterpart. Specifically, the non-uniform method has a stable and relatively constant error for a wide range of working distances and target lengths. Additionally, comparing Figures 9 and 10, we see the non-linear method reduces measurement error significantly. To better quantify this, the range of working distance was divided into separate intervals. Average and standard deviation of error, and magnitude (*i*.*e*. absolute value) of the error for both, uniform and non-uniform methods, were calculated in each interval. Table 4 presents the results. Based on this table we see another advantage of the non-uniform approach. The average value of error in the non-uniform method is almost zero; therefore, measurement error using the non-uniform approach has a random nature. Thus, multiple radial measurements can reduce the error significantly. Conversely, the average error in the uniform approach is not zero, indicating the systematic nature of the error. Finally, based on Table 4 the error of the uniform method is on average 395-times higher than the non-uniform approach. This result confirms a recent finding suggesting the presence of significant errors in horizontal measurements if nonlinear distortion of fiberoptic endoscopy is not compensated [14].

### 3.3. Experiment 3

**Figure 11.**
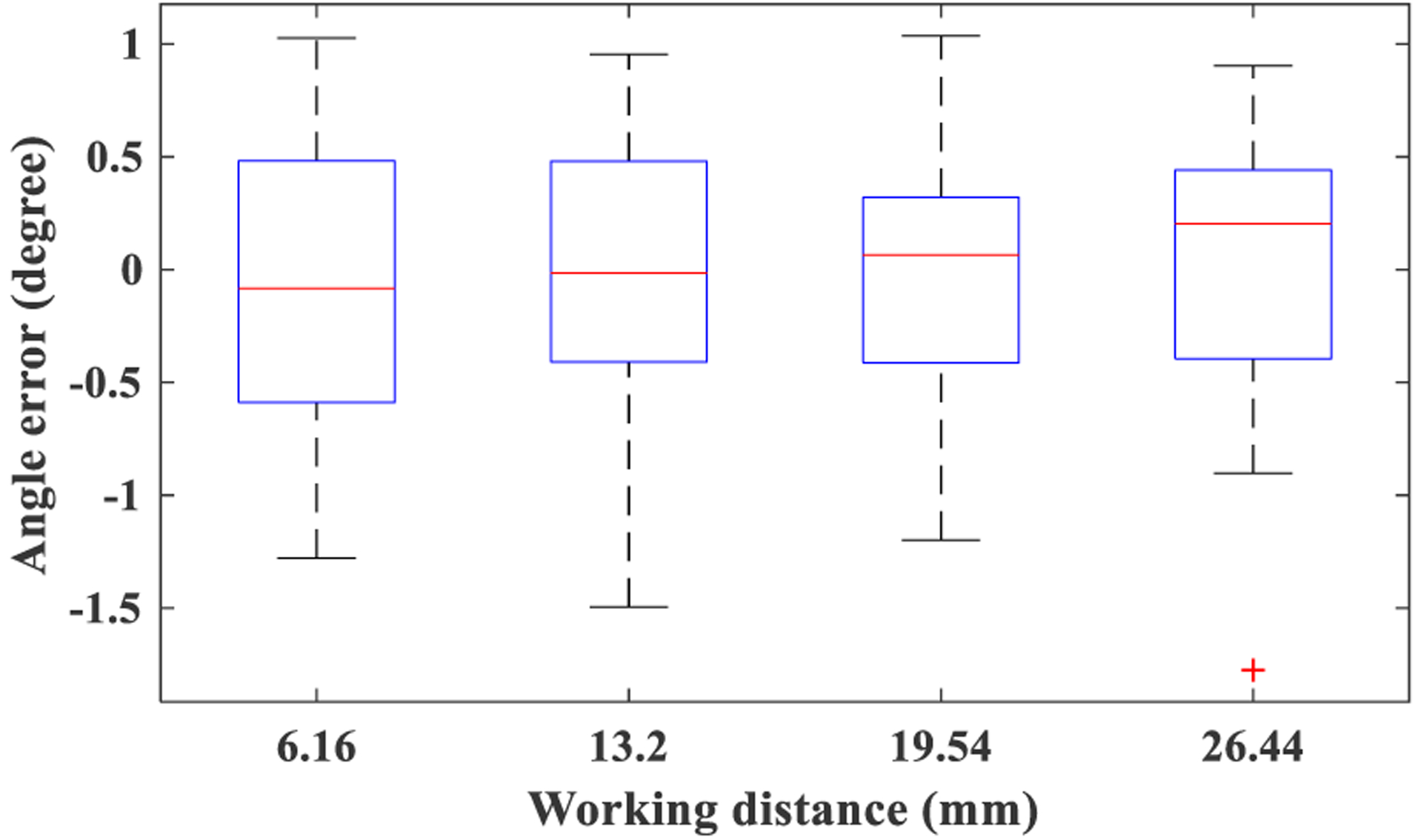
Boxplot of angle estimation error computed from set3.

This experiment was based on Set 3. Equation 8 is at the core of general calibrated measurements using the non-uniform model and relies on the angle α. Experiment 3 was conducted to investigate the accuracy of the estimation of α from an image. This experiment is especially important, given the presence of non-linear distortion in flexible endoscopy [14]. Angle differences between adjacent lines from Set 3 (Figure 3) were estimated, and then they were subtracted from their true value (*i*.*e*. 15°). Figure 11 presents boxplots of this error for different working distances. Running a one-way analysis of variance (ANOVA) did not indicate any significant effect of working distance. Therefore, all measurement errors were combined into a single group. The overall angle estimation error had the value of −0.03° ± 0.6° (average±std). Consequently, central angles can accurately be estimated from acquired images. This result may seem contradictory with a previous finding, suggesting significant errors in the estimation of angles from flexible endoscopes [31], and hence requires further explanation. The proposed method relies on central angles; however, the work of [31] was based on a general angle. Considering the radial nature of the non-linear distortion, lines passing through the center do not experience bending and curving. Therefore, the central angles can be measured very accurately.

### 3.4. Experiment 4

This experiment was based on Set 4. Specifically, Set 4 was used to compare the accuracy of uniform and non-uniform models for general horizontal measurements. Both models were trained with all data points from Set 1. Additionally, Set 4 was recorded in the presence of laser points. Therefore, the required working distance was estimated using the improved PCA method (Equations 16–17).

**Figure 12.**
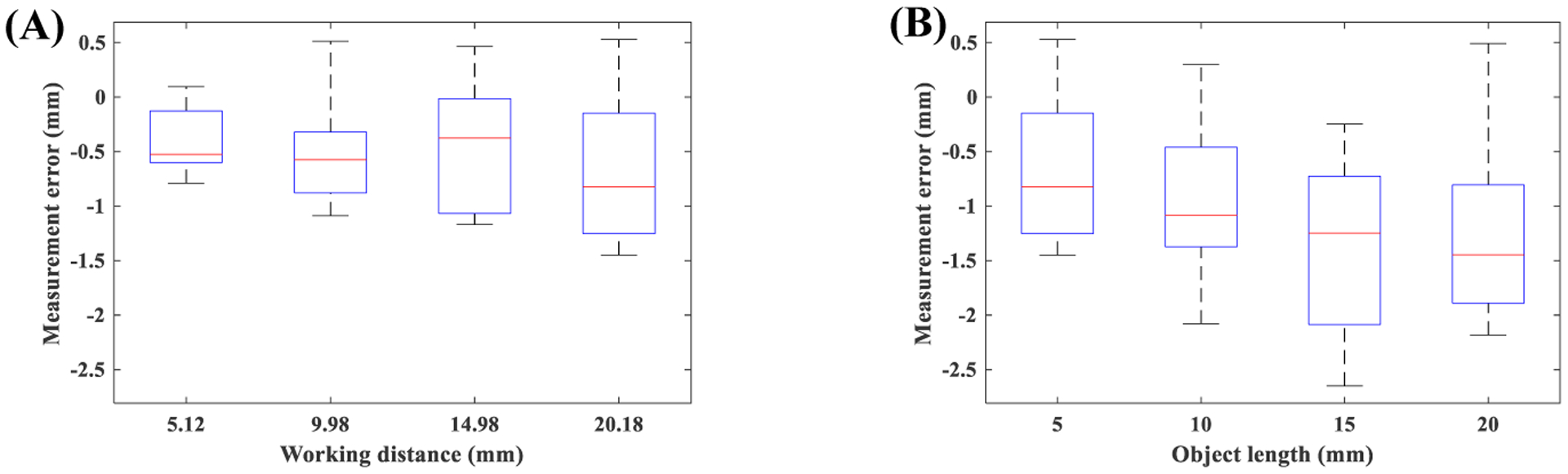
Performance of uniform model for general measurements: **(A)** effect of working distance, **(B)** effect of object length.

To investigate the effect of working distance on general horizontal measurement in the uniform model, measurement errors from a 5 mm line segment recorded at working distances of 5.12 mm, 9.98 mm, 14.98 mm, 20.18 mm were computed. One-way ANOVA with a trimming level of 0.2 and 1000 bootstrap samples [32] was non-significant (*p*=0.61). Figure 12–A presents boxplot of errors for different working distances. To investigate the effect of length of the target object on general horizontal measurement in the uniform model, measurement errors from 5 mm, 10 mm, 15 mm, 20 mm line segments recorded at the working distance of 20.18 mm were computed. One-way ANOVA with a trimming level of 0.2 and 1000 bootstrap samples [32] was non-significant (*p*=0.22). Figure 12–B presents boxplot of errors for target objects with different lengths. Considering these non-significant results, all measurement errors were combined into a single group. The overall measurement error was −0.8±0.69 mm, and the magnitude of error was 0.86±0.6 mm for the uniform method.

**Figure 13.**
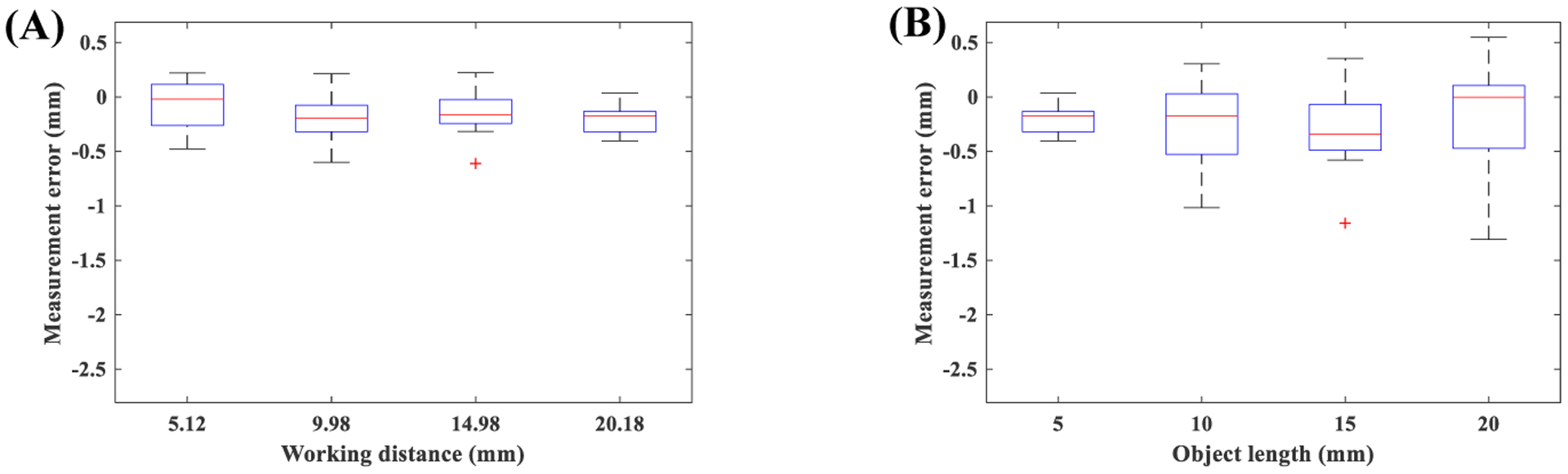
Performance of non-uniform model for general measurements: **(A)** effect of working distance, **(B)** effect of object length.

A similar approach was followed for the non-uniform method. Figure 13 presents boxplot of errors for this analysis. The effects of working distance (*p*=0.64) and length of the target object (*p*=0.43) were non-significant. Considering these non-significant results, all measurement errors were combined into a single group. The overall measurement error for the non-uniform method was −0.2±0.29 mm, and the magnitude of error was 0.27±0.24 mm.

Comparing boxplots and average errors of both methods indicate that errors from the uniform approach on average is three times higher than the non-uniform method. These results demonstrate the advantage of the proposed non-uniform approach. Investigation of boxplots of Figure 12 may indicate a general trend for errors of the uniform method. Specifically, the measurement error seems to increase with the working distance and length of the target object. In experiment 2 we saw a strong and positive correlation between uniform method error and these two parameters, which confirms this subjective observation. However, the objective analysis of ANOVA failed to detect a significant trend. Experiment 2 relied on the detection of circular shapes. This specific geometry enabled us to achieve sub-pixel resolution on measuring the length of target objects (*i*.*e*. radii of circles). However, experiment 4 was based on the detection of lines, which has the resolution of a pixel. Investigation of the performance of the non-uniform method also supports this. Specifically, the non-uniform method showed very week correlations in experiment 2 (Table 3). Therefore, we may expect to see a negligible trend for experiment 4, which subjective observation of Figure 13 confirms.

## 4. Discussion

The phonatory mechanism of the larynx is the primary voice production system in humans. It can be modeled as a dynamic system that takes air stream as the input and produces an acoustic signal in the output. The parameters of this dynamic system (*e*.*g*. vocal fold length, glottal configuration, stiffness, etc.) determine the relationship between its input and output. If we could measure and determine the input, the output, and the parameters of the system on calibrated scales, we would be able to express and model this dynamic system using mathematical equations. The method for measuring the input and output of this system, in particular for clinical voice assessment, has a long history [33]. The calibrated measurement of parameters of the phonatory system would help in achieving a more comprehensive physical model of voice production. This paper presented a method that can measure spatial parameters of the phonatory mechanism on a calibrated scale (*i*.*e*. mm). It is expected that prospective horizontal measurements would improve our understanding from the function of normal and disordered phonatory mechanisms. Additionally, it could enable us to derive computational models tuned to each patient and hence make reliable predictions about the likely outcome of different treatment options. This computational approach would advance precision and personalized medicine in the fields of laryngology and speech-language pathology. Calibrated horizontal measurements could also allow us to make direct evaluation of therapy efficacy (*e*.*g*. post-therapy reduction in the lesion size). The results of such prospective studies would advance evidence-based practice in the field of voice. Last but not least, calibrated measurements would allow us to better understand the sex differences and variations in the kinematics and morphology of the vocal folds [10,11] as well the effect of singing [12,13].

This paper provided the method for horizontal calibration and measurements from a laser-projection transnasal fiberoptic HSV system, followed by a detailed analysis of its performance in different conditions and scenarios. Flexible endoscopy images have significant non-linear distortions, which leads to the dependence of the pixel length of an object on its spatial location [14]. A previous study established the radial symmetry of this distortion [14]; hence, the proposed calibration protocol was based on circular grids. It is noteworthy that the radial nature of the distortion of flexible endoscope is also supported by the fact that the shape of circular grids is retained in the acquired images (refer to section 2.3). The proposed non-uniform method has the potency of capturing and quantifying the effects of both working distance and spatial location simultaneously. To demonstrate the efficacy of the proposed method, its performance was contrasted with a uniform approach, which assumed the independence of the pixel size of an image from its spatial location. Such uniform model is the basis of most existing methods for horizontal measurements, including all parallel laser projection systems [24].

The conducted experiments revealed several significant advantages for the non-uniform approach over its alternative uniform counterpart. Specifically, analysis of Figures 9 and 10 showed that the accuracy of radial measurements (experiment 2) using the non-uniform method was less dependent on the length of the target object and the working distance. For example, based on Table 4 we see the average magnitude of error in the non-uniform case does not change significantly when working distance increases from 5 mm to 30 mm. However, the average magnitude of error in the uniform case shows an increase of 600%. The average±std magnitude of error in uniform approach over the range of tested working distance was 0.68±0.45 mm. The average±std magnitude of error in the non-uniform approach over a similar range of working distance was 0.03±0.03 mm which further highlights the advantage of the proposed non-uniform method.

Evaluation of both methods in general measurement scenario (experiment 4) showed trends similar to radial measurements. Specifically, Figure 12 indicates that the accuracy of the uniform approach degrades with an increase in the length of the target object, whereas Figure 13 does not show any trends for the non-uniform approach. When the length of the target object increases, it spans a wider spatial location in the FOV. Considering that non-linear distortion of flexible endoscopy is spatially-dependent [14], this may translate into a larger distortion of the final image. Therefore, we may expect to see a length-dependent error for the uniform approach. It is noteworthy that this dependence did not reach the significance level, which could be attributed to the small sample size and low spatial resolution of images. Average±std magnitude of errors in the general measurement scenario resulted in 0.27±0.24 mm for non-uniform and 0.86±0.6 mm for uniform method, which is 318% higher than the non-uniform method.

Flexible endoscopes often get very close to the target object and in that sense, their typical working distance is very short. To compensate for this short working distance, flexible endoscopes rely on wide-angle lenses. Wide-angle lenses can significantly increase the size of the FOV at the expense of significant non-linear distortion in the acquired images. The above-mentioned 318% reduction in the error demonstrates the effectiveness of the proposed method for handling and compensating the introduced non-linear distortion of flexible endoscopy. Considering that other endoscopic procedures (*e*.*g*. colonoscopy, gastroendoscopy, etc.) also rely on flexible endoscopes, and the importance of calibrated measurements for medicine in general, the significance of the proposed method seems to go beyond laryngology and could easily be applied to other endoscopic procedures.

## 5. Conclusions

This work was motivated by the importance of performing calibrated (*i*.*e*. mm) spatial measurements of the vocal folds and the surrounding laryngeal structures during phonation. Such measurements would improve our understanding of the normal and disordered phonatory mechanisms and enable us to derive more accurate computational models. It is expected that evidence-based practice and precision medicine would benefit significantly from this line of research. Unfortunately, the size of a target object in laryngeal images may depend on confounding factors, which prevents calibrated spatial measurements. This study investigated the effects of two confounding factors, namely the working distance and the spatial location of the target object. To that end, a set of circular grids were recorded at multiple working distances. These grids provided an efficient way of quantifying the effect of both factors. The information from these recordings was then used to train a statistical model that would take the spatial location and the working distance of the target object as the input, and estimate the calibrated length of the target object as the output. A laser projection fiberoptic endoscope was used to estimate the working distance from the positions of the laser points. The performance of the proposed method was investigated in different scenarios. The method was also compared with a uniform model approach, where the effect of spatial location is not considered. The overall measurement error from the proposed method was −0.2±0.29 mm, and the magnitude of error was 0.27±0.24 mm. These errors were more than three times lower than the uniform model approach.

## Figures and Tables

**Table 1.** Summary of the datasets used in this study.

Dataset	Data number	Purpose	Recording specs
Set 1	65	Training the model for conversion from pixel into mm	Circular grids
			No laser light
Set 2	72	Training the model for estimation of the working distance	White paper
			With laser light
Set 3	4	Checking validity of central angle measurement from endoscopic images	15° sectors
			No laser light
Set 4	105	Evaluating accuracy of horizontal measurements	Line segments
			With laser light

**Table 2.** Correlation coefficients of uniform model for radial measurement error. The symbol ε denotes a *p*<0.0001.

	Pearson’s		Kendall’s		Spearman’s	
Parameter	r	*p*	τ	*p*	*ρ*	*p*
**Radial length**	0.59	ε	0.56	ε	0.69	ε
**Working distance**	0.76	ε	0.57	ε	0.74	ε

**Table 3.** Correlation coefficients of non-uniform model for radial measurement error. The symbol ε denotes a *p*<0.0001.

	Pearson		Kendall		Spearman	
Parameter	r	*p*	τ	*p*	*ρ*	*p*
**Radial length**	0.16	ε	0.12	ε	0.17	ε
**Working distance**	−0.14	ε	−0.08	0.003	−0.13	0.001

**Table 4.** Accuracy of radial measurements from uniform and non-uniform models in different ranges of working distance.

Working distance interval (mm)	Non-uniform				Uniform			
	Error		Magnitude of error		Error		Magnitude of error	
	mean (mm)	std (mm)	mean (mm)	std (mm)	mean (mm)	std (mm)	mean (mm)	std (mm)
**(0, 5)**	0.003	0.039	0.029	0.026	−0.192	0.077	0.192	0.075
**[5, 10)**	−0.012	0.049	0.04	0.031	−0.351	0.151	0.352	0.15
**[10, 15)**	0.02	0.028	0.025	0.024	−0.489	0.217	0.492	0.21
**[15, 20)**	−0.005	0.02	0.015	0.015	−0.692	0.303	0.693	0.299
**[20, 25)**	0.001	0.031	0.022	0.022	−0.955	0.352	0.956	0.347
**[25, 30)**	−0.001	0.039	0.029	0.026	−1.159	0.476	1.162	0.47

## References

[1] Döllinger M; Gómez P; Patel RR; Alexiou C; Bohr C; Schützenberger A Biomechanical simulation of vocal fold dynamics in adults based on laryngeal high-speed videoendoscopy. PLoS One 2017, 12, e0187486. 29121085 10.1371/journal.pone.0187486 PMC5679561

[2] Dollinger M; Hoppe U; Hettlich F; Lohscheller J; Schuberth S; Eysholdt U Vibration parameter extraction from endoscopic image series of the vocal folds. IEEE Trans. Biomed. Eng 2002, 49, 773–781. 12148815 10.1109/TBME.2002.800755

[3] Tao C; Zhang Y; Jiang JJ Extracting physiologically relevant parameters of vocal folds from high-speed video image series. IEEE Trans. Biomed. Eng 2007, 54, 794–801. 17518275 10.1109/TBME.2006.889182

[4] Behrman A Common practices of voice therapists in the evaluation of patients. J. Voice 2005, 19, 454–469. 16102671 10.1016/j.jvoice.2004.08.004

[5] Bonilha HS; Focht KL; Martin-Harris B Rater methodology for stroboscopy: a systematic review. J. Voice 2015, 29, 101–108. 25261957 10.1016/j.jvoice.2014.06.014 PMC4293207

[6] Roy N; Barkmeier-Kraemer J; Eadie T; Sivasankar MP; Mehta D; Paul D; Hillman R Evidence-based clinical voice assessment: a systematic review. Am. J. Speech-Language Pathol 2013, 22, 212–226. 10.1044/1058-0360(2012/12-0014) 23184134

[7] Patel RR; Eadie T; Paul D; Hillman R; Barkmeier-Kraemer J; Awan SN; Courey M; Deliyski D; Švec JG Recommended Protocols for Instrumental Assessment of Voice: American Speech-Language-Hearing Association Expert Panel to Develop a Protocol for Instrumental Assessment of Vocal Function. Am. J. Speech-Language Pathol 2018, 27, 887–905, doi:10.1044/2018_ajslp-17-0009. 29955816

[8] Kobler JB; Rosen DI; Burns JA; Akst LM; Broadhurst MS; Zeitels SM; Hillman RE Comparison of a flexible laryngoscope with calibrated sizing function to intraoperative measurements. Ann. Otol. Rhinol. Laryngol 2006, 115, 733–740. 17076094 10.1177/000348940611501004

[9] Patel RR; Donohue KD; Lau D; Unnikrishnan H In vivo measurement of pediatric vocal fold motion using structured light laser projection. J. Voice 2013, 27, 463–472. 23809569 10.1016/j.jvoice.2013.03.004 PMC3772768

[10] Hamdan A-L; Khalifee E; Ziade G; Semaan S Sexual Dimorphism in Laryngeal Volumetric Measurements Using Magnetic Resonance Imaging. Ear, Nose Throat J. 2020, 99, 132–136. 31018691 10.1177/0145561319840568

[11] Yamauchi A; Yokonishi H; Imagawa H; Sakakibara K-I; Nito T; Tayama N; Yamasoba T Age- and gender-related difference of vocal fold vibration and glottal configuration in normal speakers: analysis with glottal area waveform. J. Voice 2014, 28, 525–531. 24836359 10.1016/j.jvoice.2014.01.016

[12] Roers F; Mürbe D; Sundberg J Predicted singers’ vocal fold lengths and voice classification a study of x-ray morphological measures. J. Voice 2009, 23, 408–413. 18395418 10.1016/j.jvoice.2007.12.003

[13] Unteregger F; Wagner P; Honegger F; Potthast S; Zwicky S; Storck C Changes in vocal fold morphology during singing over two octaves. J. Voice 2020, 34, 165–169. 10.1016/j.jvoice.2018.08.020 30266281

[14] Ghasemzadeh H; Deliyski D Non-linear image distortions in flexible fiberoptic endoscopes and their effects on calibrated horizontal measurements. J. Voice 2020, [Epub ahead of print]. 10.1016/j.jvoice.2020.08.029 PMC7969477 32958427

[15] Schuberth S; Hoppe U; Döllinger M; Lohscheller J; Eysholdt U High-precision measurement of the vocal fold length and vibratory amplitudes. Laryngoscope 2002, 112, 1043–1049. 12160271 10.1097/00005537-200206000-00020

[16] Schade G; Leuwer R; Kraas M; Rassow B; Hess MM Laryngeal morphometry with a new laser “clip on” device. Lasers Surg. Med. Off. J. Am. Soc. Laser Med. Surg 2004, 34, 363–367. 10.1002/lsm.20065 15216528

[17] Larsson H; Hertegård S Calibration of high-speed imaging by laser triangulation. Logop. Phoniatr. Vocology 2004, 29, 154–161. 10.1080/14015430410024353 15764209

[18] Patel RR; Donohue KD; Johnson WC; Archer SM Laser projection imaging for measurement of pediatric voice. Laryngoscope 2011, 121, 2411–2417. 21993904 10.1002/lary.22325 PMC3320049

[19] Luegmair G; Mehta DD; Kobler JB; Döllinger M Three-Dimensional Optical Reconstruction of Vocal Fold Kinematics Using High-Speed Video With a Laser Projection System. IEEE Trans. Med. Imaging 2015, 34, 2572–2582. 26087485 10.1109/TMI.2015.2445921 PMC4666755

[20] Deliyski DD; Shishkov M; Mehta DD; Ghasemzadeh H; Bouma B; Zañartu M; de Alarcon A; Hillman RE Laser-Calibrated System for Transnasal Fiberoptic Laryngeal High-Speed Videoendoscopy. J. Voice 2021, 35, 122–128. 31383516 10.1016/j.jvoice.2019.07.013 PMC6995434

[21] Maguluri G; Mehta D; Kobler J; Park J; Iftimia N Synchronized, concurrent optical coherence tomography and videostroboscopy for monitoring vocal fold morphology and kinematics. Biomed. Opt. Express 2019, 10, 4450–4461. 31565501 10.1364/BOE.10.004450 PMC6757476

[22] Coughlan CA; Chou L; Jing JC; Chen JJ; Rangarajan S; Chang TH; Sharma GK; Cho K; Lee D; Goddard JA; In vivo cross-sectional imaging of the phonating larynx using long-range Doppler optical coherence tomography. Sci. Rep 2016, 6, 22792. 26960250 10.1038/srep22792 PMC4785353

[23] Burns JA Optical coherence tomography: imaging the larynx. Curr. Opin. Otolaryngol. Head Neck Surg 2012, 20, 477–481. 22913932 10.1097/MOO.0b013e3283582d7d

[24] Ghasemzadeh H; Deliyski D; Ford D; Kobler JB; Hillman RE; Mehta DD Method for Vertical Calibration of Laser-Projection Transnasal Fiberoptic High-Speed Videoendoscopy. J. Voice 2020, 34, 847–861. 10.1016/j.jvoice.2019.04.015 PMC6883161 31151853

[25] Herzon GD; Zealear DL New laser ruler instrument for making measurements through an endoscope. Otolaryngol. Neck Surg 1997, 116, 689–692. 10.1016/S0194-5998(97)70251-1 29389262

[26] Wurzbacher T; Voigt I; Schwarz R; Döllinger M; Hoppe U; Penne J; Eysholdt U; Lohscheller J Calibration of laryngeal endoscopic high-speed image sequences by an automated detection of parallel laser line projections. Med. Image Anal 2008, 12, 300–317. 18373942 10.1016/j.media.2007.12.007

[27] Luegmair G; Kniesburges S; Zimmermann M; Sutor A; Eysholdt U; Dollinger M Optical reconstruction of high-speed surface dynamics in an uncontrollable environment. IEEE Trans. Med. Imaging 2010, 29, 1979–1991. 21118756 10.1109/TMI.2010.2055578

[28] Semmler M; Kniesburges S; Birk V; Ziethe A; Patel R; Döllinger M 3D reconstruction of human laryngeal dynamics based on endoscopic high-speed recordings. IEEE Trans. Med. Imaging 2016, 35, 1615–1624. 26829782 10.1109/TMI.2016.2521419

[29] Smith WJ; Smith WJ Modern optical engineering; 3rd, Ed.; Mcgraw-hill New York, 2000;

[30] Duda RO; Hart PE Use of the Hough transformation to detect lines and curves in pictures. Commun. ACM 1972, 15, 11–15.

[31] Dailey SH; Kobler JB; Hillman RE; Tangrom K; Thananart E; Mauri M; Zeitels SM Endoscopic measurement of vocal fold movement during adduction and abduction. Laryngoscope 2005, 115, 178–183. 15630390 10.1097/01.mlg.0000150701.46377.df

[32] Wilcox RR Introduction to robust estimation and hypothesis testing; Academic press, 2011;

[33] Baken RJ; Orlikoff RF Clinical measurement of speech and voice; Cengage Learning, 2000;

